# 18 High Mobility Group Box-1 Is Not Correlated with the Persistent Hyperinflammatory Response After Burn Injury

**DOI:** 10.1093/jbcr/irae036.018

**Published:** 2024-04-17

**Authors:** Desiree Pinto, Tuan D Le, Anthony Pusateri, Shane Matthew, Melissa M McLawhorn, Lauren T Moffatt, Jeffrey W Shupp

**Affiliations:** MedStar Washington Hospital Center, Washington, DC; MedStar Washington Hospital Center, Grand Prairie, Texas; Naval Medical Research Unit San Antonio, JBSA-Fort Sam Houston, San Antonio, Texas; MedStar Burn Research Lab, Washington, DC; MedStar Health Research Institute, Washington, DC; Firefighters’ Burn and Surgical Research Laboratory, Washington, DC; Georgetown University School of Medicine, Washington, DC; MedStar Washington Hospital Center, Washington, DC; MedStar Washington Hospital Center, Grand Prairie, Texas; Naval Medical Research Unit San Antonio, JBSA-Fort Sam Houston, San Antonio, Texas; MedStar Burn Research Lab, Washington, DC; MedStar Health Research Institute, Washington, DC; Firefighters’ Burn and Surgical Research Laboratory, Washington, DC; Georgetown University School of Medicine, Washington, DC; MedStar Washington Hospital Center, Washington, DC; MedStar Washington Hospital Center, Grand Prairie, Texas; Naval Medical Research Unit San Antonio, JBSA-Fort Sam Houston, San Antonio, Texas; MedStar Burn Research Lab, Washington, DC; MedStar Health Research Institute, Washington, DC; Firefighters’ Burn and Surgical Research Laboratory, Washington, DC; Georgetown University School of Medicine, Washington, DC; MedStar Washington Hospital Center, Washington, DC; MedStar Washington Hospital Center, Grand Prairie, Texas; Naval Medical Research Unit San Antonio, JBSA-Fort Sam Houston, San Antonio, Texas; MedStar Burn Research Lab, Washington, DC; MedStar Health Research Institute, Washington, DC; Firefighters’ Burn and Surgical Research Laboratory, Washington, DC; Georgetown University School of Medicine, Washington, DC; MedStar Washington Hospital Center, Washington, DC; MedStar Washington Hospital Center, Grand Prairie, Texas; Naval Medical Research Unit San Antonio, JBSA-Fort Sam Houston, San Antonio, Texas; MedStar Burn Research Lab, Washington, DC; MedStar Health Research Institute, Washington, DC; Firefighters’ Burn and Surgical Research Laboratory, Washington, DC; Georgetown University School of Medicine, Washington, DC; MedStar Washington Hospital Center, Washington, DC; MedStar Washington Hospital Center, Grand Prairie, Texas; Naval Medical Research Unit San Antonio, JBSA-Fort Sam Houston, San Antonio, Texas; MedStar Burn Research Lab, Washington, DC; MedStar Health Research Institute, Washington, DC; Firefighters’ Burn and Surgical Research Laboratory, Washington, DC; Georgetown University School of Medicine, Washington, DC; MedStar Washington Hospital Center, Washington, DC; MedStar Washington Hospital Center, Grand Prairie, Texas; Naval Medical Research Unit San Antonio, JBSA-Fort Sam Houston, San Antonio, Texas; MedStar Burn Research Lab, Washington, DC; MedStar Health Research Institute, Washington, DC; Firefighters’ Burn and Surgical Research Laboratory, Washington, DC; Georgetown University School of Medicine, Washington, DC

## Abstract

**Introduction:**

Severe burn injury results in a hyperinflammatory response, that has been linked to mortality. Damage-associated molecular patterns (DAMPS), endogenous molecules released during cell necrosis/apoptosis, are released into circulation immediately after burn injury and contribute to the upregulation of the immune system. The change in DAMP levels and their continued impact on the immune system after burn injury is less known. This study aims to evaluate the evolution of high mobility group box 1 (HMGB1), a specific DAMP, after burn injury and its role in the development of a persistent hyperinflammatory state.

**Methods:**

This was a prospective observational study that included patients admitted to a regional burn center. Blood samples to measure HMGB1 and cytokine protein levels (IL-1, IL-6, IL-10, IL-12, TNF-a) were collected at admission and at set time intervals up to 21 days after admission. The patients were divided into groups based on mortality status. Differences in HMGB1 levels between groups were compared by using a two-sample t-test or Wilcoxon rank-sum test and chi-square or Fisher exact test for data at admission and a mixed-effect model for repeated measures. HMGB1 levels were correlated to cytokine protein levels using Pearson Correlation Coefficient. A p-value of < 0.05 signified statistical significance.

**Results:**

There were 115 patients with both HMGB1 and cytokine protein levels out of the 158 patients enrolled. Overall mortality was 12.9%, median TBSA was 11.8%, and median age was 40. At admission, the HMGB1 level was significantly higher for patients who died than survived (>10ng/mL vs 6.2ng/mL). For all patients the peak level of HMGB1 was at admission and then markedly decreased over the next 48 hours. A significant difference between patients who died and lived persisted for 48 hours (4.4ng/mL vs 2.9ng/mL). At admission, all cytokine protein levels were significantly elevated. Over the next 48-72 hours, the cytokine protein levels continued to increase for IL-1, IL-6, IL-12, and TNF alpha, and decreased for IL-10. At admission, there was a low positive correlation between HMGB1 and IL-6 (0.21), IL-10 (0.25), and TNF-alpha (0.26), and no positive correlation after admission (p < 0.05).

**Conclusions:**

Mortality was associated with significantly higher levels of a HMGB1 at admission and up to 48 hours after injury. While HMGB1 and other DAMPS may trigger the initial hyperinflammatory response, their contribution to the persistent hyperinflammatory response following burn injury is less clear.

**Applicability of Research to Practice:**

HMGB1, a specific DAMP, has some utility in predicting mortality and serving as a biomarker for inflammation at admission. However, HMGB1 may not be the most appropriate biomarker to determine the development of a persistent hyperinflammatory state in the burn population.

